# INTERVENTIONS TO REDUCE STIGMA OF DEMENTIA: FINDINGS FROM A SCOPING REVIEW

**DOI:** 10.1093/geroni/igz038.1729

**Published:** 2019-11-08

**Authors:** Juanita R Bacsu, Marc Viger, Shanthi Johnson, Tom McIntosh, Bonnie Jeffery, Nuelle Novik, Paul Hackett

**Affiliations:** 1 University of Regina, Regina, Saskatchewan, Canada; 2 University of Saskatchewan, Saskatoon, Saskatchewan, Canada; 3 University of Alberta, Edmonton, Alberta, Canada

## Abstract

Although there is significant stigma attached to dementia, there is a paucity of knowledge on stigma reduction interventions. Guided by a strength-based approach, this presentation consists of two objectives: 1) to identify the literature on interventions to reduce dementia-related stigma; and 2) to recognize the strength-based components of existing anti-stigma interventions. A five-stage scoping review process was used to examine peer-reviewed literature of anti-stigma interventions of dementia from 2008 to 2018. From 744 initial records, 21 articles matched our inclusion criteria and were reviewed. A stigma reduction framework was used for classifying interventions: education (to dispel myths with accurate information), contact (to provide interaction with people with dementia), mixed (education and contact interventions), and protest (to challenge negative attitudes of dementia). A range of education, contact, and mixed interventions were identified. Strength-based components of education interventions included using: facts to dispel myths, multiple mediums to support dementia information, and culturally-informed strategies for specific audiences. Key components of contact and mixed interventions included: showcasing the achievements of people with dementia, relationship-building, and engaging in purposeful learning. Findings from this study can help to inform future interventions to reduce stigma and improve the quality of life for people affected by dementia.

